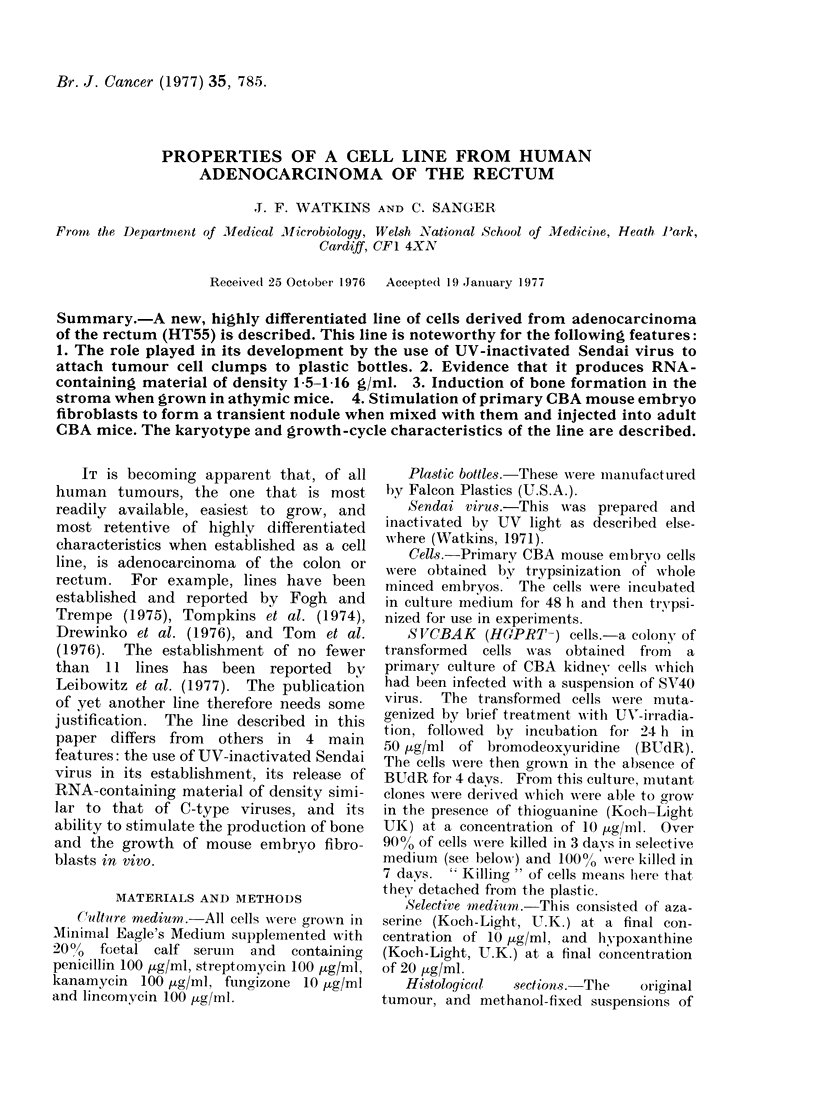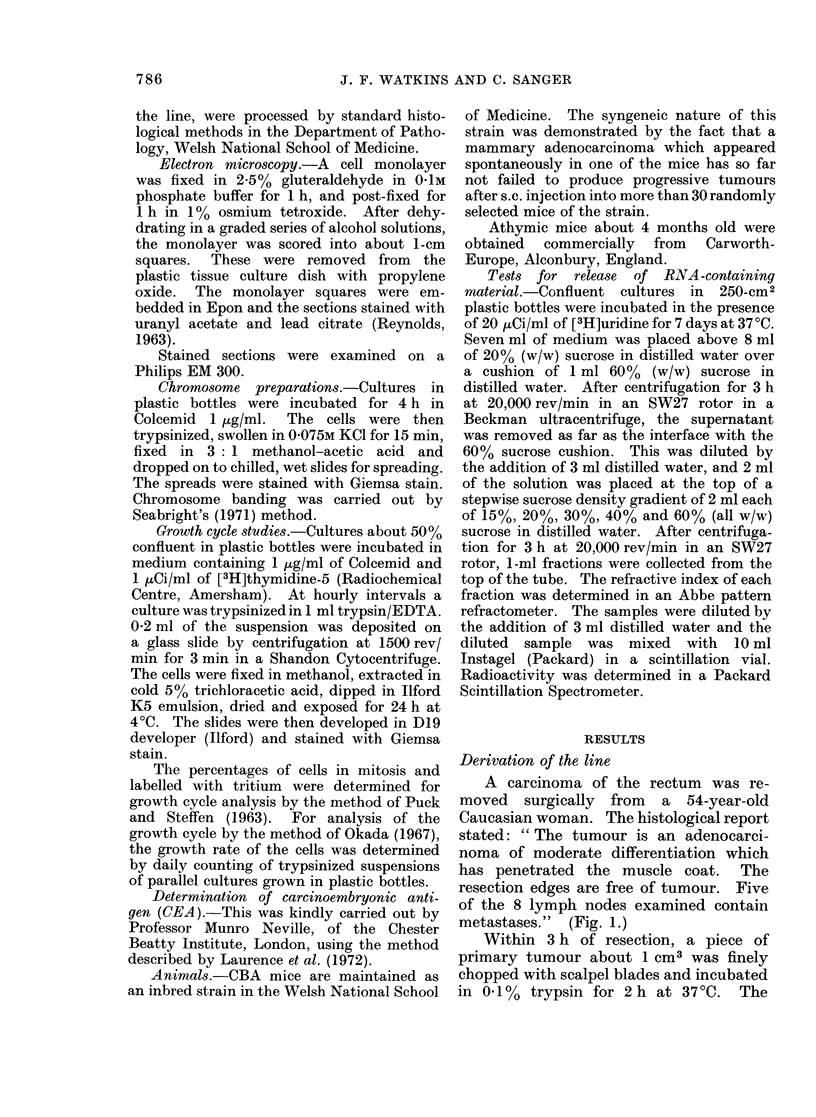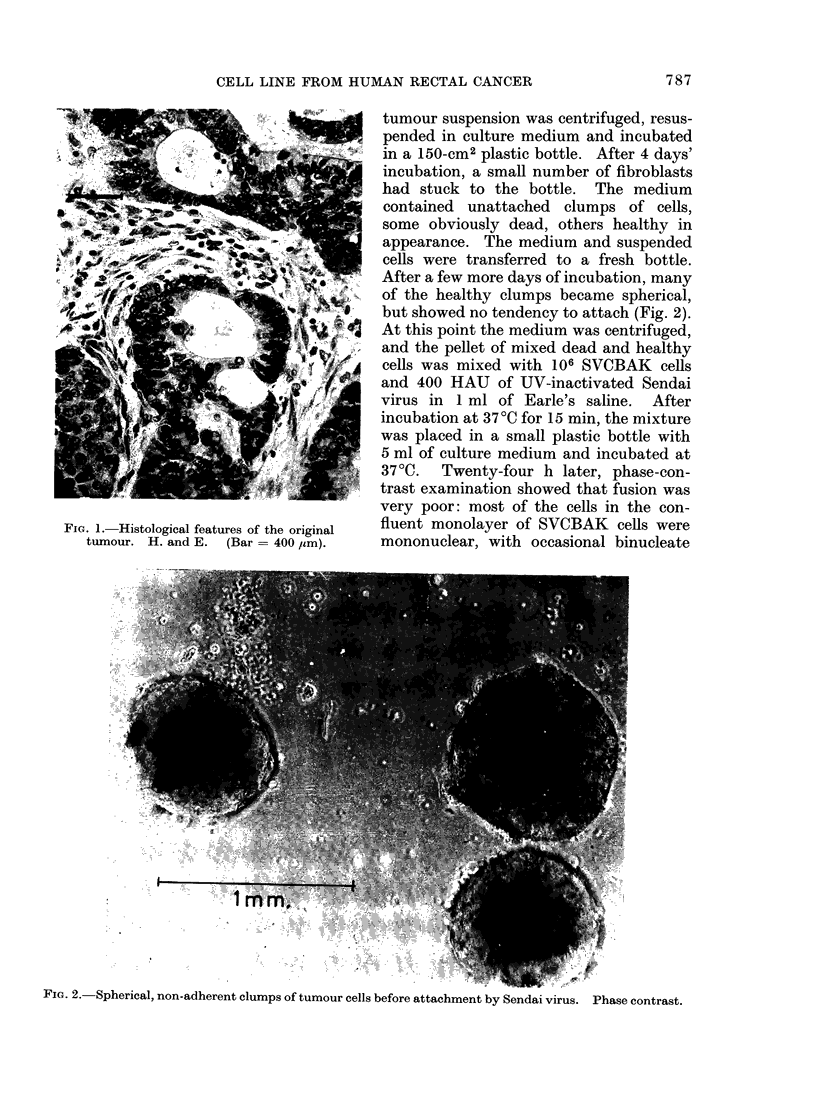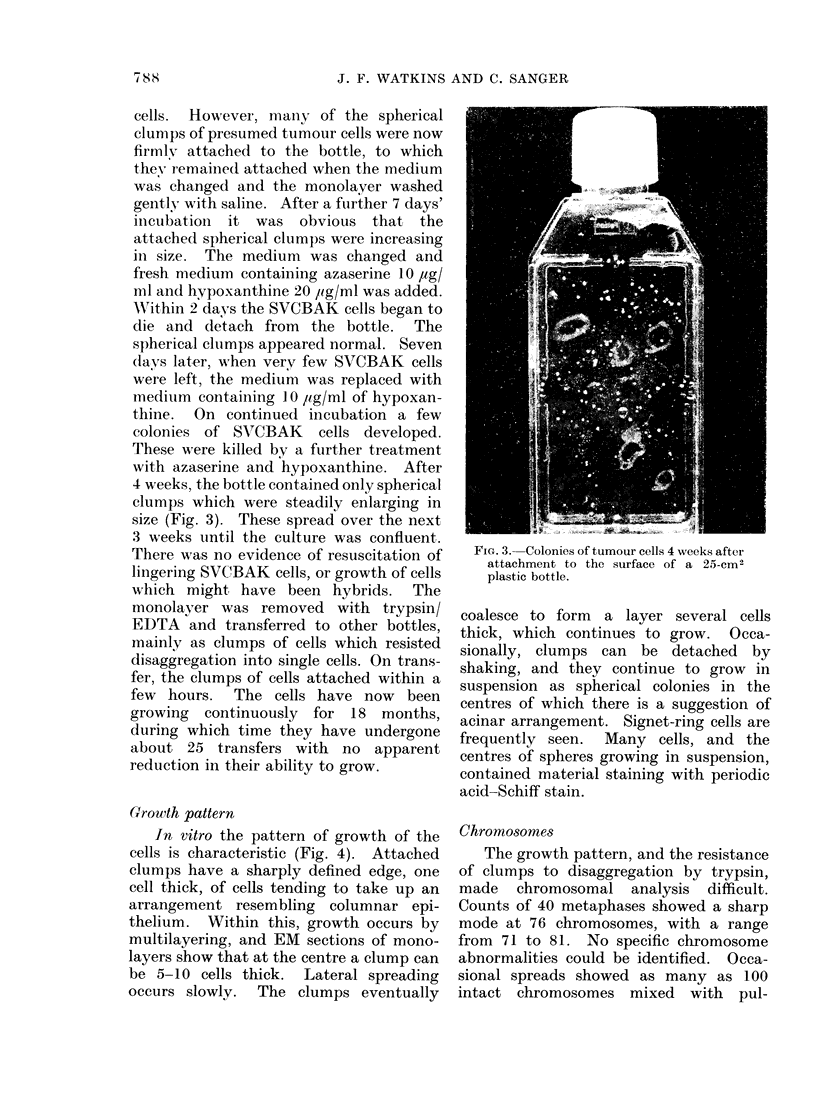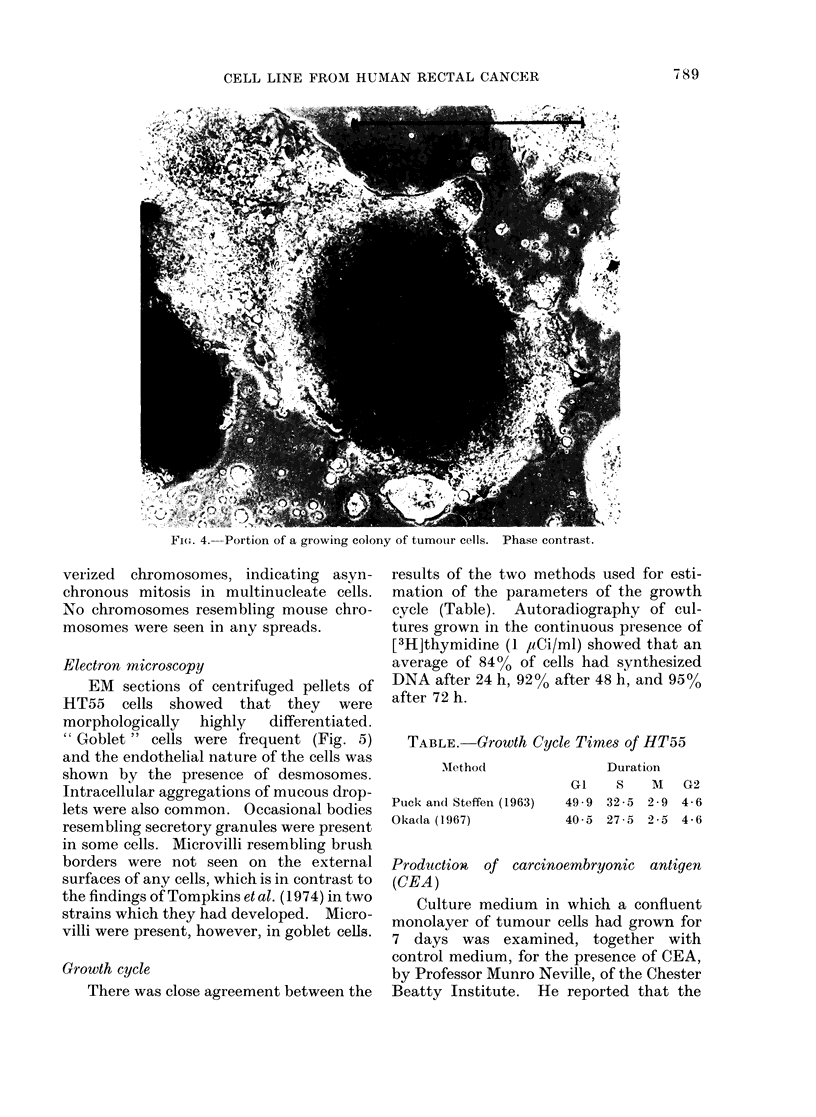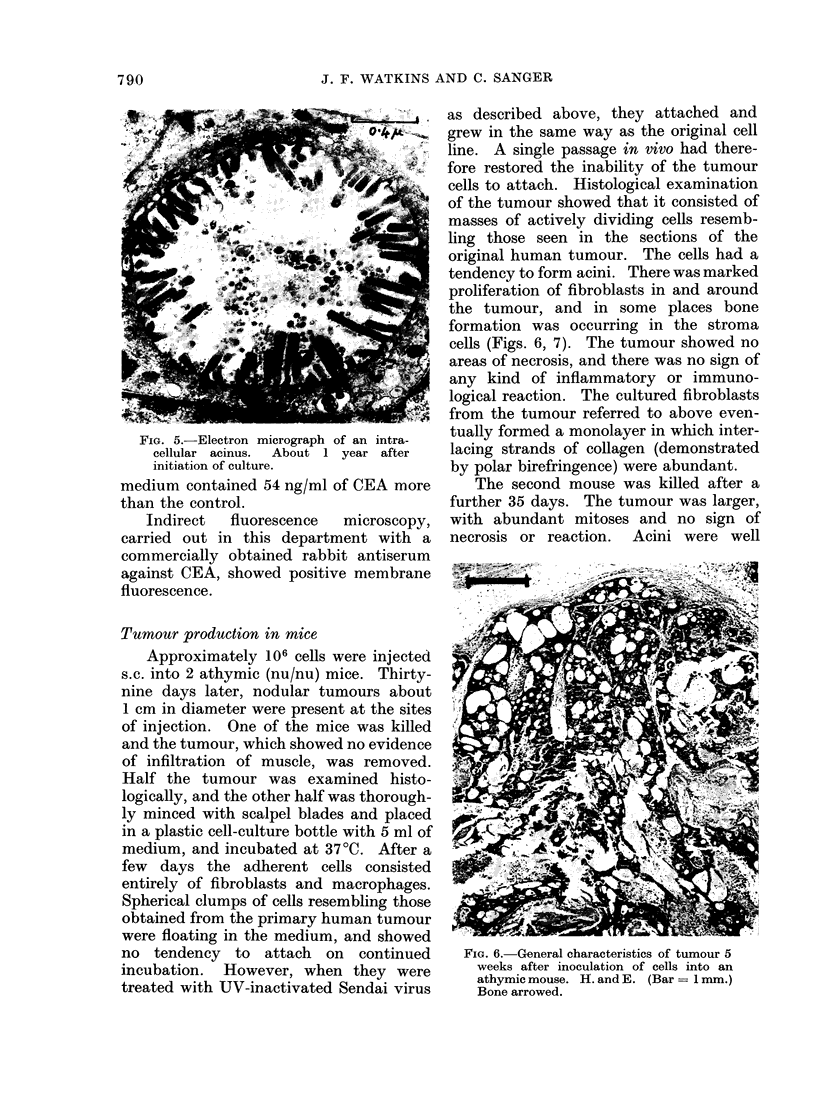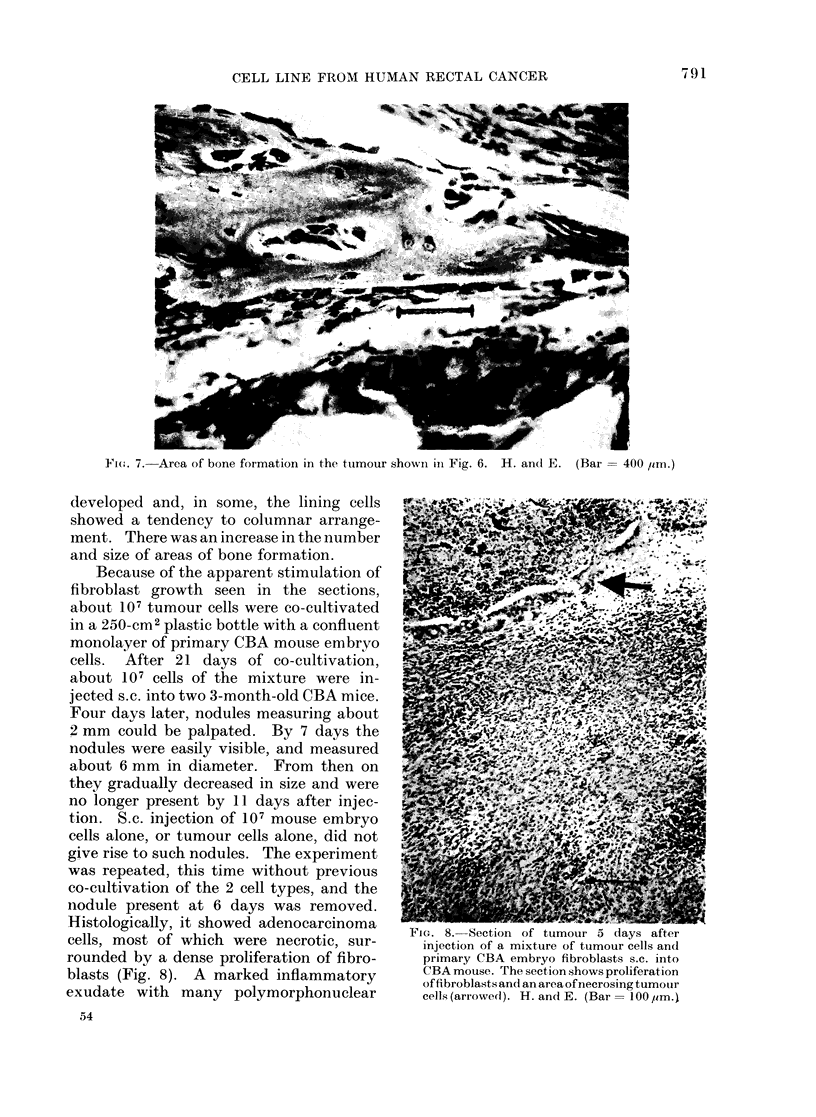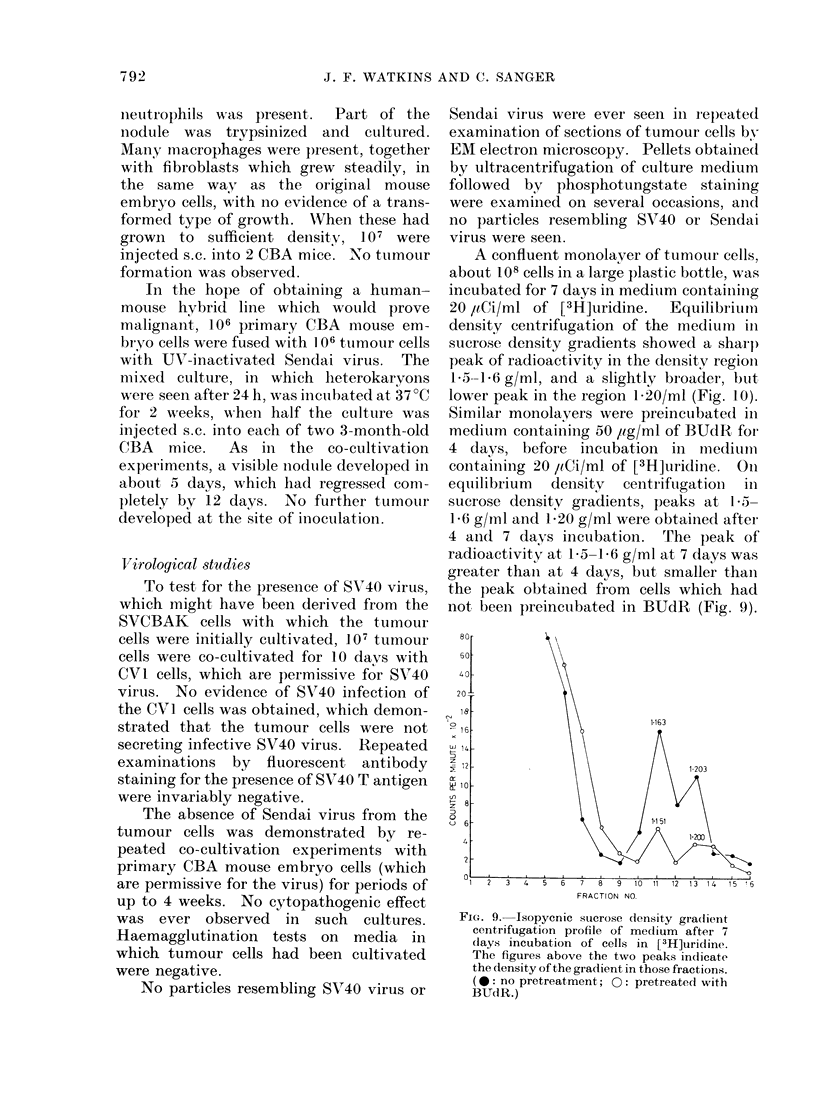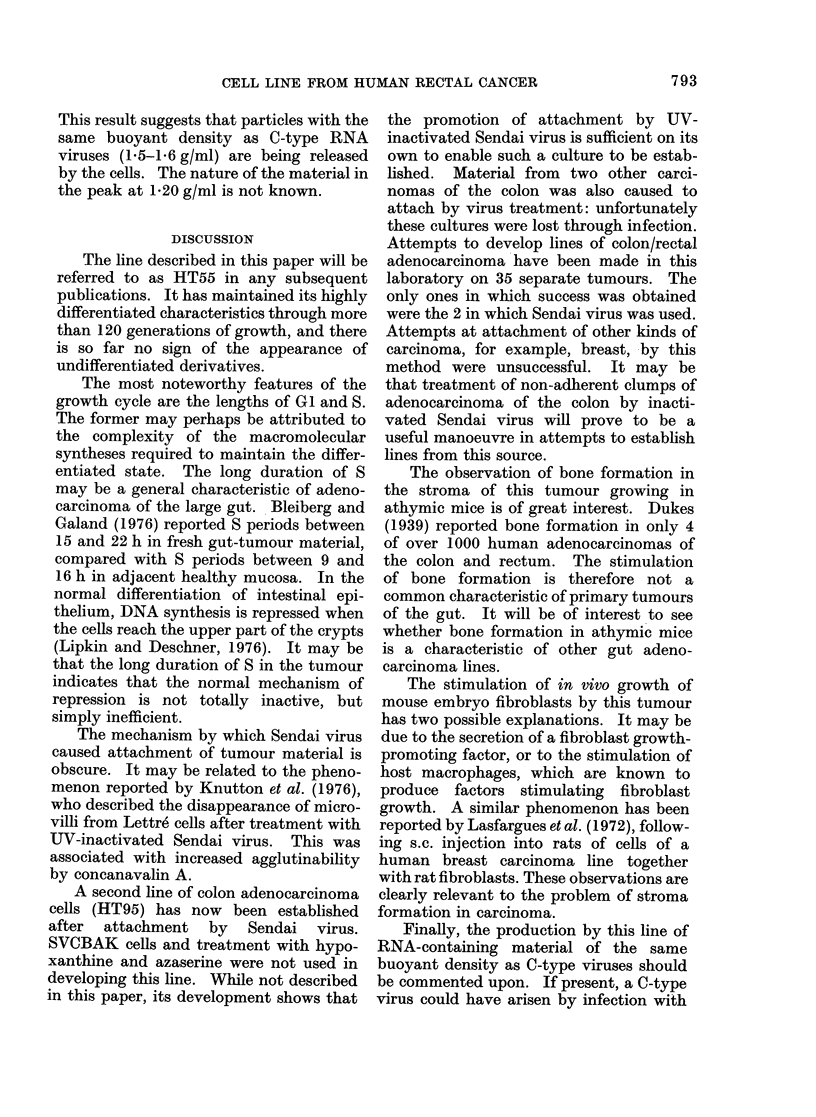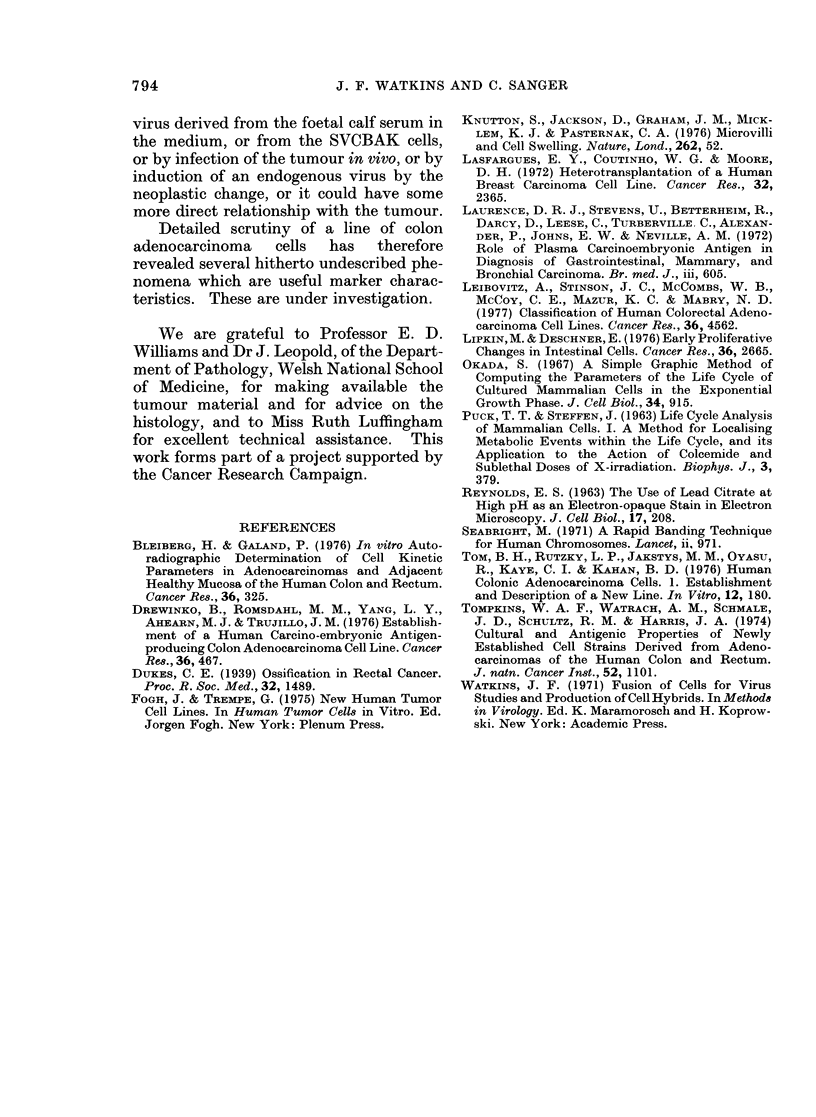# Properties of a cell line from human adenocarcinoma of the rectum.

**DOI:** 10.1038/bjc.1977.120

**Published:** 1977-06

**Authors:** J. F. Watkins, C. Sanger

## Abstract

**Images:**


*Br. J. Cancer* (1977) **35**, 785.

### PROPERTIES OF A CELL LINE FROM HUMAN ADENOCARCINOMA OF THE RECTUM

J. F. WATKINS AND C. SANGER

*From the Department of Medical Microbiology, Welsh National School of Medicine, Heath Park, Cardiff, CF1 4XN*

Received 25 October 1976 Accepted 19 January 1977

**Summary.—A new, highly differentiated line of cells derived from adenocarcinoma of the rectum (HT55) is described. This line is noteworthy for the following features: 1. The role played in its development by the use of UV-inactivated Sendai virus to attach tumour cell clumps to plastic bottles. 2. Evidence that it produces RNA-containing material of density 1·5–1·16 g/ml. 3. Induction of bone formation in the stroma when grown in athymic mice. 4. Stimulation of primary CBA mouse embryo fibroblasts to form a transient nodule when mixed with them and injected into adult CBA mice. The karyotype and growth-cycle characteristics of the line are described.**

It is becoming apparent that, of all human tumours, the one that is most readily available, easiest to grow, and most retentive of highly differentiated characteristics when established as a cell line, is adenocarcinoma of the colon or rectum. For example, lines have been established and reported by Fogh and Trempe (1975), Tompkins *et al.* (1974), Drewinko *et al.* (1976), and Tom *et al.* (1976). The establishment of no fewer than 11 lines has been reported by Leibowitz *et al.* (1977). The publication of yet another line therefore needs some justification. The line described in this paper differs from others in 4 main features: the use of UV-inactivated Sendai virus in its establishment, its release of RNA-containing material of density similar to that of C-type viruses, and its ability to stimulate the production of bone and the growth of mouse embryo fibroblasts *in vivo*.

### MATERIALS AND METHODS

*Culture medium.*—All cells were grown in Minimal Eagle's Medium supplemented with 20% foetal calf serum and containing penicillin 100 μg/ml, streptomycin 100 μg/ml, kanamycin 100 μg/ml, fungizone 10 μg/ml and lincomycin 100 μg/ml.

*Plastic bottles.*—These were manufactured by Falcon Plastics (U.S.A.).

*Sendai virus.*—This was prepared and inactivated by UV light as described elsewhere (Watkins, 1971).

*Cells.*—Primary CBA mouse embryo cells were obtained by trypsinization of whole minced embryos. The cells were incubated in culture medium for 48 h and then trypsinized for use in experiments.

*SVCBAK* ($HGPRT^-$) cells.—a colony of transformed cells was obtained from a primary culture of CBA kidney cells which had been infected with a suspension of SV40 virus. The transformed cells were mutagenized by brief treatment with UV-irradiation, followed by incubation for 24 h in 50 μg/ml of bromodeoxyuridine (BUdR). The cells were then grown in the absence of BUdR for 4 days. From this culture, mutant clones were derived which were able to grow in the presence of thioguanine (Koch–Light UK) at a concentration of 10 μg/ml. Over 90% of cells were killed in 3 days in selective medium (see below) and 100% were killed in 7 days. " Killing " of cells means here that they detached from the plastic.

*Selective medium.*—This consisted of azaserine (Koch-Light, U.K.) at a final concentration of 10 μg/ml, and hypoxanthine (Koch-Light, U.K.) at a final concentration of 20 μg/ml.

*Histological sections.*—The original tumour, and methanol-fixed suspensions of the line, were processed by standard histological methods in the Department of Pathology, Welsh National School of Medicine.

*Electron microscopy.*—A cell monolayer was fixed in 2·5% gluteraldehyde in 0·1M phosphate buffer for 1 h, and post-fixed for 1 h in 1% osmium tetroxide. After dehydrating in a graded series of alcohol solutions, the monolayer was scored into about 1-cm squares. These were removed from the plastic tissue culture dish with propylene oxide. The monolayer squares were embedded in Epon and the sections stained with uranyl acetate and lead citrate (Reynolds, 1963).

Stained sections were examined on a Philips EM 300.

*Chromosome preparations.*—Cultures in plastic bottles were incubated for 4 h in Colcemid 1 μg/ml. The cells were then trypsinized, swollen in 0·075M KCl for 15 min, fixed in 3 : 1 methanol–acetic acid and dropped on to chilled, wet slides for spreading. The spreads were stained with Giemsa stain. Chromosome banding was carried out by Seabright's (1971) method.

*Growth cycle studies.*—Cultures about 50% confluent in plastic bottles were incubated in medium containing 1 μg/ml of Colcemid and 1 μCi/ml of [$^3$H]thymidine-5 (Radiochemical Centre, Amersham). At hourly intervals a culture was trypsinized in 1 ml trypsin/EDTA. 0·2 ml of the suspension was deposited on a glass slide by centrifugation at 1500 rev/min for 3 min in a Shandon Cytocentrifuge. The cells were fixed in methanol, extracted in cold 5% trichloracetic acid, dipped in Ilford K5 emulsion, dried and exposed for 24 h at 4°C. The slides were then developed in D19 developer (Ilford) and stained with Giemsa stain.

The percentages of cells in mitosis and labelled with tritium were determined for growth cycle analysis by the method of Puck and Steffen (1963). For analysis of the growth cycle by the method of Okada (1967), the growth rate of the cells was determined by daily counting of trypsinized suspensions of parallel cultures grown in plastic bottles.

*Determination of carcinoembryonic antigen (CEA).*—This was kindly carried out by Professor Munro Neville, of the Chester Beatty Institute, London, using the method described by Laurence *et al.* (1972).

*Animals.*—CBA mice are maintained as an inbred strain in the Welsh National School of Medicine. The syngeneic nature of this strain was demonstrated by the fact that a mammary adenocarcinoma which appeared spontaneously in one of the mice has so far not failed to produce progressive tumours after s.c. injection into more than 30 randomly selected mice of the strain.

Athymic mice about 4 months old were obtained commercially from Carworth-Europe, Alconbury, England.

*Tests for release of RNA-containing material.*—Confluent cultures in 250-cm$^2$ plastic bottles were incubated in the presence of 20 μCi/ml of [$^3$H]uridine for 7 days at 37°C. Seven ml of medium was placed above 8 ml of 20% (w/w) sucrose in distilled water over a cushion of 1 ml 60% (w/w) sucrose in distilled water. After centrifugation for 3 h at 20,000 rev/min in an SW27 rotor in a Beckman ultracentrifuge, the supernatant was removed as far as the interface with the 60% sucrose cushion. This was diluted by the addition of 3 ml distilled water, and 2 ml of the solution was placed at the top of a stepwise sucrose density gradient of 2 ml each of 15%, 20%, 30%, 40% and 60% (all w/w) sucrose in distilled water. After centrifugation for 3 h at 20,000 rev/min in an SW27 rotor, 1-ml fractions were collected from the top of the tube. The refractive index of each fraction was determined in an Abbe pattern refractometer. The samples were diluted by the addition of 3 ml distilled water and the diluted sample was mixed with 10 ml Instagel (Packard) in a scintillation vial. Radioactivity was determined in a Packard Scintillation Spectrometer.

### RESULTS

### *Derivation of the line*

A carcinoma of the rectum was removed surgically from a 54-year-old Caucasian woman. The histological report stated: "The tumour is an adenocarcinoma of moderate differentiation which has penetrated the muscle coat. The resection edges are free of tumour. Five of the 8 lymph nodes examined contain metastases." (Fig. 1.)

Within 3 h of resection, a piece of primary tumour about 1 cm$^3$ was finely chopped with scalpel blades and incubated in 0·1% trypsin for 2 h at 37°C. The

FIG. 1.—Histological features of the original tumour. H. and E. (Bar = 400 μm).

tumour suspension was centrifuged, resuspended in culture medium and incubated in a 150-cm² plastic bottle. After 4 days' incubation, a small number of fibroblasts had stuck to the bottle. The medium contained unattached clumps of cells, some obviously dead, others healthy in appearance. The medium and suspended cells were transferred to a fresh bottle. After a few more days of incubation, many of the healthy clumps became spherical, but showed no tendency to attach (Fig. 2). At this point the medium was centrifuged, and the pellet of mixed dead and healthy cells was mixed with $10^6$ SVCBAK cells and 400 HAU of UV-inactivated Sendai virus in 1 ml of Earle's saline. After incubation at 37 °C for 15 min, the mixture was placed in a small plastic bottle with 5 ml of culture medium and incubated at 37 °C. Twenty-four h later, phase-contrast examination showed that fusion was very poor: most of the cells in the confluent monolayer of SVCBAK cells were mononuclear, with occasional binucleate

FIG. 2.—Spherical, non-adherent clumps of tumour cells before attachment by Sendai virus. Phase contrast.

cells. However, many of the spherical clumps of presumed tumour cells were now firmly attached to the bottle, to which they remained attached when the medium was changed and the monolayer washed gently with saline. After a further 7 days' incubation it was obvious that the attached spherical clumps were increasing in size. The medium was changed and fresh medium containing azaserine 10 $\mu$g/ml and hypoxanthine 20 $\mu$g/ml was added. Within 2 days the SVCBAK cells began to die and detach from the bottle. The spherical clumps appeared normal. Seven days later, when very few SVCBAK cells were left, the medium was replaced with medium containing 10 $\mu$g/ml of hypoxanthine. On continued incubation a few colonies of SVCBAK cells developed. These were killed by a further treatment with azaserine and hypoxanthine. After 4 weeks, the bottle contained only spherical clumps which were steadily enlarging in size (Fig. 3). These spread over the next 3 weeks until the culture was confluent. There was no evidence of resuscitation of lingering SVCBAK cells, or growth of cells which might have been hybrids. The monolayer was removed with trypsin/EDTA and transferred to other bottles, mainly as clumps of cells which resisted disaggregation into single cells. On transfer, the clumps of cells attached within a few hours. The cells have now been growing continuously for 18 months, during which time they have undergone about 25 transfers with no apparent reduction in their ability to grow.

FIG. 3.—Colonies of tumour cells 4 weeks after attachment to the surface of a 25-cm² plastic bottle.

*Growth pattern*

*In vitro* the pattern of growth of the cells is characteristic (Fig. 4). Attached clumps have a sharply defined edge, one cell thick, of cells tending to take up an arrangement resembling columnar epithelium. Within this, growth occurs by multilayering, and EM sections of monolayers show that at the centre a clump can be 5–10 cells thick. Lateral spreading occurs slowly. The clumps eventually coalesce to form a layer several cells thick, which continues to grow. Occasionally, clumps can be detached by shaking, and they continue to grow in suspension as spherical colonies in the centres of which there is a suggestion of acinar arrangement. Signet-ring cells are frequently seen. Many cells, and the centres of spheres growing in suspension, contained material staining with periodic acid–Schiff stain.

*Chromosomes*

The growth pattern, and the resistance of clumps to disaggregation by trypsin, made chromosomal analysis difficult. Counts of 40 metaphases showed a sharp mode at 76 chromosomes, with a range from 71 to 81. No specific chromosome abnormalities could be identified. Occasional spreads showed as many as 100 intact chromosomes mixed with pul-

FIG. 4.—Portion of a growing colony of tumour cells. Phase contrast.

verized chromosomes, indicating asynchronous mitosis in multinucleate cells. No chromosomes resembling mouse chromosomes were seen in any spreads.

*Electron microscopy*

EM sections of centrifuged pellets of HT55 cells showed that they were morphologically highly differentiated. " Goblet " cells were frequent (Fig. 5) and the endothelial nature of the cells was shown by the presence of desmosomes. Intracellular aggregations of mucous droplets were also common. Occasional bodies resembling secretory granules were present in some cells. Microvilli resembling brush borders were not seen on the external surfaces of any cells, which is in contrast to the findings of Tompkins *et al.* (1974) in two strains which they had developed. Microvilli were present, however, in goblet cells.

*Growth cycle*

There was close agreement between the results of the two methods used for estimation of the parameters of the growth cycle (Table). Autoradiography of cultures grown in the continuous presence of [$^3$H]thymidine (1 $\mu$Ci/ml) showed that an average of 84% of cells had synthesized DNA after 24 h, 92% after 48 h, and 95% after 72 h.

TABLE.—*Growth Cycle Times of HT*55

| Method | Duration | | | |
|---|---|---|---|---|
| | G1 | S | M | G2 |
| Puck and Steffen (1963) | 49·9 | 32·5 | 2·9 | 4·6 |
| Okada (1967) | 40·5 | 27·5 | 2·5 | 4·6 |

*Production of carcinoembryonic antigen (CEA)*

Culture medium in which a confluent monolayer of tumour cells had grown for 7 days was examined, together with control medium, for the presence of CEA, by Professor Munro Neville, of the Chester Beatty Institute. He reported that the

FIG. 5.—Electron micrograph of an intracellular acinus. About 1 year after initiation of culture.

medium contained 54 ng/ml of CEA more than the control.

Indirect fluorescence microscopy, carried out in this department with a commercially obtained rabbit antiserum against CEA, showed positive membrane fluorescence.

*Tumour production in mice*

Approximately $10^6$ cells were injected s.c. into 2 athymic (nu/nu) mice. Thirty-nine days later, nodular tumours about 1 cm in diameter were present at the sites of injection. One of the mice was killed and the tumour, which showed no evidence of infiltration of muscle, was removed. Half the tumour was examined histologically, and the other half was thoroughly minced with scalpel blades and placed in a plastic cell-culture bottle with 5 ml of medium, and incubated at 37 °C. After a few days the adherent cells consisted entirely of fibroblasts and macrophages. Spherical clumps of cells resembling those obtained from the primary human tumour were floating in the medium, and showed no tendency to attach on continued incubation. However, when they were treated with UV-inactivated Sendai virus as described above, they attached and grew in the same way as the original cell line. A single passage *in vivo* had therefore restored the inability of the tumour cells to attach. Histological examination of the tumour showed that it consisted of masses of actively dividing cells resembling those seen in the sections of the original human tumour. The cells had a tendency to form acini. There was marked proliferation of fibroblasts in and around the tumour, and in some places bone formation was occurring in the stroma cells (Figs. 6, 7). The tumour showed no areas of necrosis, and there was no sign of any kind of inflammatory or immunological reaction. The cultured fibroblasts from the tumour referred to above eventually formed a monolayer in which interlacing strands of collagen (demonstrated by polar birefringence) were abundant.

The second mouse was killed after a further 35 days. The tumour was larger, with abundant mitoses and no sign of necrosis or reaction. Acini were well

FIG. 6.—General characteristics of tumour 5 weeks after inoculation of cells into an athymic mouse. H. and E. (Bar = 1 mm.) Bone arrowed.

FIG. 7.—Area of bone formation in the tumour shown in Fig. 6. H. and E. (Bar = 400 $\mu$m.)

developed and, in some, the lining cells showed a tendency to columnar arrangement. There was an increase in the number and size of areas of bone formation.

Because of the apparent stimulation of fibroblast growth seen in the sections, about $10^7$ tumour cells were co-cultivated in a 250-cm$^2$ plastic bottle with a confluent monolayer of primary CBA mouse embryo cells. After 21 days of co-cultivation, about $10^7$ cells of the mixture were injected s.c. into two 3-month-old CBA mice. Four days later, nodules measuring about 2 mm could be palpated. By 7 days the nodules were easily visible, and measured about 6 mm in diameter. From then on they gradually decreased in size and were no longer present by 11 days after injection. S.c. injection of $10^7$ mouse embryo cells alone, or tumour cells alone, did not give rise to such nodules. The experiment was repeated, this time without previous co-cultivation of the 2 cell types, and the nodule present at 6 days was removed. Histologically, it showed adenocarcinoma cells, most of which were necrotic, surrounded by a dense proliferation of fibroblasts (Fig. 8). A marked inflammatory exudate with many polymorphonuclear

FIG. 8.—Section of tumour 5 days after injection of a mixture of tumour cells and primary CBA embryo fibroblasts s.c. into CBA mouse. The section shows proliferation of fibroblasts and an area of necrosing tumour cells (arrowed). H. and E. (Bar = 100 $\mu$m.)

neutrophils was present. Part of the nodule was trypsinized and cultured. Many macrophages were present, together with fibroblasts which grew steadily, in the same way as the original mouse embryo cells, with no evidence of a transformed type of growth. When these had grown to sufficient density, $10^7$ were injected s.c. into 2 CBA mice. No tumour formation was observed.

In the hope of obtaining a human–mouse hybrid line which would prove malignant, $10^6$ primary CBA mouse embryo cells were fused with $10^6$ tumour cells with UV-inactivated Sendai virus. The mixed culture, in which heterokaryons were seen after 24 h, was incubated at 37 °C for 2 weeks, when half the culture was injected s.c. into each of two 3-month-old CBA mice. As in the co-cultivation experiments, a visible nodule developed in about 5 days, which had regressed completely by 12 days. No further tumour developed at the site of inoculation.

*Virological studies*

To test for the presence of SV40 virus, which might have been derived from the SVCBAK cells with which the tumour cells were initially cultivated, $10^7$ tumour cells were co-cultivated for 10 days with CV1 cells, which are permissive for SV40 virus. No evidence of SV40 infection of the CV1 cells was obtained, which demonstrated that the tumour cells were not secreting infective SV40 virus. Repeated examinations by fluorescent antibody staining for the presence of SV40 T antigen were invariably negative.

The absence of Sendai virus from the tumour cells was demonstrated by repeated co-cultivation experiments with primary CBA mouse embryo cells (which are permissive for the virus) for periods of up to 4 weeks. No cytopathogenic effect was ever observed in such cultures. Haemagglutination tests on media in which tumour cells had been cultivated were negative.

No particles resembling SV40 virus or Sendai virus were ever seen in repeated examination of sections of tumour cells by EM electron microscopy. Pellets obtained by ultracentrifugation of culture medium followed by phosphotungstate staining were examined on several occasions, and no particles resembling SV40 or Sendai virus were seen.

A confluent monolayer of tumour cells, about $10^8$ cells in a large plastic bottle, was incubated for 7 days in medium containing 20 $\mu$Ci/ml of [$^3$H]uridine. Equilibrium density centrifugation of the medium in sucrose density gradients showed a sharp peak of radioactivity in the density region 1·5–1·6 g/ml, and a slightly broader, but lower peak in the region 1·20/ml (Fig. 10). Similar monolayers were preincubated in medium containing 50 $\mu$g/ml of BUdR for 4 days, before incubation in medium containing 20 $\mu$Ci/ml of [$^3$H]uridine. On equilibrium density centrifugation in sucrose density gradients, peaks at 1·5–1·6 g/ml and 1·20 g/ml were obtained after 4 and 7 days incubation. The peak of radioactivity at 1·5–1·6 g/ml at 7 days was greater than at 4 days, but smaller than the peak obtained from cells which had not been preincubated in BUdR (Fig. 9).

FIG. 9.—Isopycnic sucrose density gradient centrifugation profile of medium after 7 days incubation of cells in [$^3$H]uridine. The figures above the two peaks indicate the density of the gradient in those fractions. (●: no pretreatment; ○: pretreated with BUdR.)

This result suggests that particles with the same buoyant density as C-type RNA viruses (1·5–1·6 g/ml) are being released by the cells. The nature of the material in the peak at 1·20 g/ml is not known.

### DISCUSSION

The line described in this paper will be referred to as HT55 in any subsequent publications. It has maintained its highly differentiated characteristics through more than 120 generations of growth, and there is so far no sign of the appearance of undifferentiated derivatives.

The most noteworthy features of the growth cycle are the lengths of G1 and S. The former may perhaps be attributed to the complexity of the macromolecular syntheses required to maintain the differentiated state. The long duration of S may be a general characteristic of adenocarcinoma of the large gut. Bleiberg and Galand (1976) reported S periods between 15 and 22 h in fresh gut-tumour material, compared with S periods between 9 and 16 h in adjacent healthy mucosa. In the normal differentiation of intestinal epithelium, DNA synthesis is repressed when the cells reach the upper part of the crypts (Lipkin and Deschner, 1976). It may be that the long duration of S in the tumour indicates that the normal mechanism of repression is not totally inactive, but simply inefficient.

The mechanism by which Sendai virus caused attachment of tumour material is obscure. It may be related to the phenomenon reported by Knutton *et al.* (1976), who described the disappearance of microvilli from Lettré cells after treatment with UV-inactivated Sendai virus. This was associated with increased agglutinability by concanavalin A.

A second line of colon adenocarcinoma cells (HT95) has now been established after attachment by Sendai virus. SVCBAK cells and treatment with hypoxanthine and azaserine were not used in developing this line. While not described in this paper, its development shows that the promotion of attachment by UV-inactivated Sendai virus is sufficient on its own to enable such a culture to be established. Material from two other carcinomas of the colon was also caused to attach by virus treatment: unfortunately these cultures were lost through infection. Attempts to develop lines of colon/rectal adenocarcinoma have been made in this laboratory on 35 separate tumours. The only ones in which success was obtained were the 2 in which Sendai virus was used. Attempts at attachment of other kinds of carcinoma, for example, breast, by this method were unsuccessful. It may be that treatment of non-adherent clumps of adenocarcinoma of the colon by inactivated Sendai virus will prove to be a useful manoeuvre in attempts to establish lines from this source.

The observation of bone formation in the stroma of this tumour growing in athymic mice is of great interest. Dukes (1939) reported bone formation in only 4 of over 1000 human adenocarcinomas of the colon and rectum. The stimulation of bone formation is therefore not a common characteristic of primary tumours of the gut. It will be of interest to see whether bone formation in athymic mice is a characteristic of other gut adenocarcinoma lines.

The stimulation of *in vivo* growth of mouse embryo fibroblasts by this tumour has two possible explanations. It may be due to the secretion of a fibroblast growth-promoting factor, or to the stimulation of host macrophages, which are known to produce factors stimulating fibroblast growth. A similar phenomenon has been reported by Lasfargues *et al.* (1972), following s.c. injection into rats of cells of a human breast carcinoma line together with rat fibroblasts. These observations are clearly relevant to the problem of stroma formation in carcinoma.

Finally, the production by this line of RNA-containing material of the same buoyant density as C-type viruses should be commented upon. If present, a C-type virus could have arisen by infection with virus derived from the foetal calf serum in the medium, or from the SVCBAK cells, or by infection of the tumour *in vivo*, or by induction of an endogenous virus by the neoplastic change, or it could have some more direct relationship with the tumour.

Detailed scrutiny of a line of colon adenocarcinoma cells has therefore revealed several hitherto undescribed phenomena which are useful marker characteristics. These are under investigation.

We are grateful to Professor E. D. Williams and Dr J. Leopold, of the Department of Pathology, Welsh National School of Medicine, for making available the tumour material and for advice on the histology, and to Miss Ruth Luffingham for excellent technical assistance. This work forms part of a project supported by the Cancer Research Campaign.

### REFERENCES

BLEIBERG, H. & GALAND, P. (1976) *In vitro* Autoradiographic Determination of Cell Kinetic Parameters in Adenocarcinomas and Adjacent Healthy Mucosa of the Human Colon and Rectum. *Cancer Res.*, **36**, 325.

DREWINKO, B., ROMSDAHL, M. M., YANG, L. Y., AHEARN, M. J. & TRUJILLO, J. M. (1976) Establishment of a Human Carcino-embryonic Antigen-producing Colon Adenocarcinoma Cell Line. *Cancer Res.*, **36**, 467.

DUKES, C. E. (1939) Ossification in Rectal Cancer. *Proc. R. Soc. Med.*, **32**, 1489.

FOGH, J. & TREMPE, G. (1975) New Human Tumor Cell Lines. In *Human Tumor Cells* in Vitro. Ed. Jorgen Fogh. New York: Plenum Press.

KNUTTON, S., JACKSON, D., GRAHAM, J. M., MICKLEM, K. J. & PASTERNAK, C. A. (1976) Microvilli and Cell Swelling. *Nature, Lond.*, **262**, 52.

LASFARGUES, E. Y., COUTINHO, W. G. & MOORE, D. H. (1972) Heterotransplantation of a Human Breast Carcinoma Cell Line. *Cancer Res.*, **32**, 2365.

LAURENCE, D. R. J., STEVENS, U., BETTERHEIM, R., DARCY, D., LEESE, C., TURBERVILLE. C., ALEXANDER, P., JOHNS, E. W. & NEVILLE, A. M. (1972) Role of Plasma Carcinoembryonic Antigen in Diagnosis of Gastrointestinal, Mammary, and Bronchial Carcinoma. *Br. med. J.*, iii, 605.

LEIBOVITZ, A., STINSON, J. C., MCCOMBS, W. B., MCCOY, C. E., MAZUR, K. C. & MABRY, N. D. (1977) Classification of Human Colorectal Adenocarcinoma Cell Lines. *Cancer Res.*, **36**, 4562.

LIPKIN, M. & DESCHNER, E. (1976) Early Proliferative Changes in Intestinal Cells. *Cancer Res.*, **36**, 2665.

OKADA, S. (1967) A Simple Graphic Method of Computing the Parameters of the Life Cycle of Cultured Mammalian Cells in the Exponential Growth Phase. *J. Cell Biol.*, **34**, 915.

PUCK, T. T. & STEFFEN, J. (1963) Life Cycle Analysis of Mammalian Cells. I. A Method for Localising Metabolic Events within the Life Cycle, and its Application to the Action of Colcemide and Sublethal Doses of X-irradiation. *Biophys. J.*, **3**, 379.

REYNOLDS, E. S. (1963) The Use of Lead Citrate at High pH as an Electron-opaque Stain in Electron Microscopy. *J. Cell Biol.*, **17**, 208.

SEABRIGHT, M. (1971) A Rapid Banding Technique for Human Chromosomes. *Lancet*, ii, 971.

TOM, B. H., RUTZKY, L. P., JAKSTYS, M. M., OYASU, R., KAYE, C. I. & KAHAN, B. D. (1976) Human Colonic Adenocarcinoma Cells. 1. Establishment and Description of a New Line. *In Vitro*, **12**, 180.

TOMPKINS, W. A. F., WATRACH, A. M., SCHMALE, J. D., SCHULTZ, R. M. & HARRIS, J. A. (1974) Cultural and Antigenic Properties of Newly Established Cell Strains Derived from Adenocarcinomas of the Human Colon and Rectum. *J. natn. Cancer Inst.*, **52**, 1101.

WATKINS, J. F. (1971) Fusion of Cells for Virus Studies and Production of Cell Hybrids. In *Methods in Virology*. Ed. K. Maramorosch and H. Koprowski. New York: Academic Press.